# Decision tree and reinforcement learning for contextual electricity consumption forecasting in buildings

**DOI:** 10.1016/j.mex.2026.104011

**Published:** 2026-06-18

**Authors:** Daniel Ramos, Pedro Faria, Pedro Campos, Zita Vale

**Affiliations:** a GECAD – Research Group on Intelligent Engineering and Computing for Advanced Innovation and Development, Polytechnic of Porto, Portugal; b FEP - School of Economics and Management, University of Porto, Portugal

**Keywords:** Energy management, Multiarmed bandit, Reinforcement learning

## Abstract

A promising opportunity to optimize energy control and storage is the use of prediction. Several forecasting algorithms from the artificial intelligence area like the Neural Networks or from the machine learning field as K-Nearest Neighbors and XGBoost are recommended for prediction tasks involving the estimation of energy patterns ahead of time. Additionally, it is recommended to apply wisely the forecasting algorithm relying on unique contexts that define different target periods.

The method in this paper targets the prediction of consumptions of a building scheduled for all periods of five minutes of a week:

• with the support of K-Nearest Neighbors and Neural Networks
• a decision tree identifies unique contexts according to rules that rely on all the patterns with energy and sensors data of a building for periods of five minutes
• a Multiarmed Bandit algorithm gifted with reinforcement learning capabilities selects the algorithm more convenient for prediction tasks.

The results and conclusions indicate that identification of contexts through decision rules results in higher confidence bounds while evaluating the most effective forecasting algorithm. The SMAPE forecasting errors obtained in the third context were 3.54% with KNN and 4.79% with ANN. The obtained SMAPE forecasting errors in the fourth context were 4.91% with KNN and 4.54% with ANN.

## Graphical abstract

**Figure:**
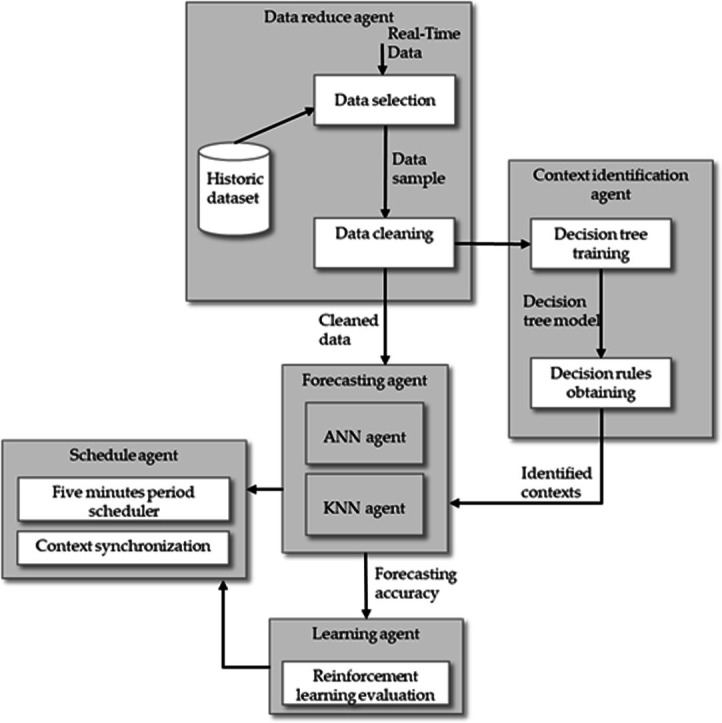

**Specifications table**

**Table:** 

**Subject area**	Energy
**More specific subject area**	Load forecasting
**Name of your method**	Hybrid Decision Tree and Reinforcement Learning for Contextual Electricity Consumption
**Name and reference of original method**	Artificial Neural Networks, K-Nearest Neighbors, Decision Tree, Multi-Armed Bandit
**Resource availability**	Python, Tensorflow/Keras, Scikit-learn, Pandas

## Background

Problems characterized by uncertainty can be addressed through decision-making mechanisms based on reinforcement learning (RL) [1]. The effectiveness of optimization strategies depends on factors such as data monitoring and the chosen RL technique [2]. However, forecasting tasks with strong temporal correlations and convergence instability remain challenging [3]. To improve prediction accuracy, methods such as decision trees and clustering algorithms can classify historical data [4], while incorporating additional variables with non-linear relationships further enhances performance [5].

Decision trees have been widely applied in forecasting and energy systems. For instance, a photovoltaic generation forecasting model combines decision trees with bootstrap aggregation and improved feature selection [6]. Similarly, decision trees support optimization in microgrid energy dispatch [7]. They can also be integrated into RL frameworks to reduce computational complexity by limiting the action space, avoiding exponential growth during decision exploration [1].

RL has demonstrated strong applicability in energy markets and management systems. Multi-agent RL improves bidding strategies in electricity markets by handling incomplete information and maximizing profits [8]. In household energy management, RL helps address uncertainties in electricity prices and consumption patterns [9]. It also enhances both global and local forecasting approaches [10], including weather prediction using hybrid RL-based methods with high temporal resolution [11]. Furthermore, RL enables optimized strategies for energy storage systems by balancing cost, capacity, and reliability [12].

In complex systems such as multi-energy residential communities, RL frameworks treat devices as individual agents, reducing computational complexity while addressing incomplete information [13]. Carefully designed reward functions ensure that RL agents continue searching for optimal solutions rather than settling for suboptimal ones [14].

RL also improves decentralized decision-making in microgrids, where agents operate independently without full knowledge of others [15]. Applications such as ARLEM demonstrate how RL can dynamically update policies to improve energy management in urban buildings [16]. Beyond this, RL plays a key role in optimizing building-integrated photovoltaic systems, particularly in high-dimensional and uncertain environments [17]. It is also applied in vehicle-to-grid systems, considering interactions among grids, aggregators, users, and scheduling uncertainties [18].

Motivated by these advancements, this work proposes a framework that combines decision trees and RL for building energy forecasting. Decision trees identify distinct operational contexts based on features such as energy consumption, sensor data (e.g., CO₂ levels and light intensity), and temporal variables. Subsequently, a multi-armed bandit RL approach selects the most suitable forecasting algorithm for each context. The study employs K-Nearest Neighbors and ANN models for training and prediction, building on prior work. Previous case study demonstrates the effectiveness of this approach in estimating building energy consumption across different time periods [19]. Moreover, in [20], we used a similar work with decision tree rules to create unique contexts and a greedy learning method that decides the most viable algorithm to predict the consumption of the building according to the period in question.

This article brings a new look to the existing literature in the following areas: (I) upper confidence bound learning method; (II) increase the decision tree accuracy; and (III) improve accuracy of load forecast. The scientific aim of the work is to find the best methods and algorithms for each context. The subject of the research was to obtain improved accuracy of load forecasting in buildings.

### Method details

**Fig. 1:**
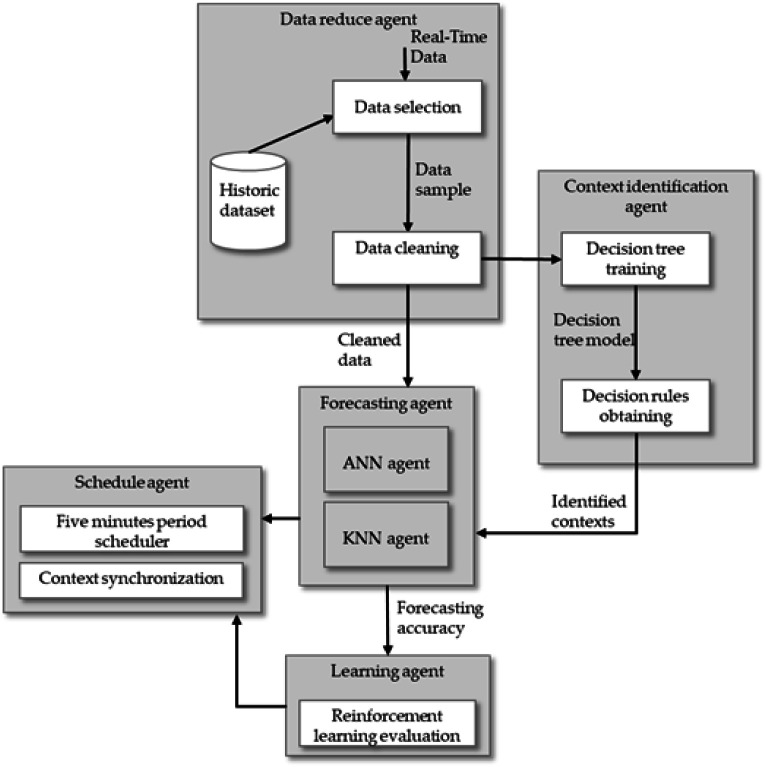
Proposed contextual approach.

This section presents the sequence of steps involved in the contextualization of the proposed approach. This approach involves four types of agents with different roles: data processing, forecasting activities, schedule and learning. Fig. 1 illustrates the specific roles of each one of the four agents and their interaction. The proposed approach includes the following pipeline:

### Decision tree → context identification → multi-armed bandit-based model selection → forecasting

Using a decision tree, several contexts are identified. Then forecasting of load is obtained with a model selected by the reinforcement learning multi-armed bandit algorithm. In fact, after cleaning the data, it is used both for decision tree in the definition of contexts, which already identify contexts and prelaminar choose of ANN or KNN, and for making the forecast using ANN and KNN. However, contexts are defined in a planning phase while the actual forecast is done for every single period. Then, finally, the learning agent applies the reinforcement learning in order to anticipate, for each next period, what should the method (ANN or KNN) to be considered.

The Schedule agent synchronizes the predictions of the consumption from Forecasting agent to periods of five minutes. Moreover, this schedule differentiates the contexts defined previously by the decision tree rules.

In the agent communications, the Data reduce agent is responsible for reducing the historic dataset to a simplified version. Firstly, a data sample is collected from the historic dataset. This sample features energy consumption from a building and sensors with a high correlation. The rules to select data from a historic dataset apply also to real-time data. Afterwards, cleaning operations delete manually unreliable consumption patterns and delete automatically detected outliers. The Context identification agent uses the resulting data that was processed by the previous agent to train a decision tree, thus obtaining decision rules which identify different contexts to perform forecasts.

The rules of the decision tree model are obtained with the support of training data with five features of the building including the day of week, period of the day, consumption, light intensity, and CO2. The training of the decision tree relies on the value of a maximum depth parameter that is the quantity of splits that the tree can perform from the root to the leaf.

**Table:** 

Pseudocode of decision tree
FOR D from 2 to N DO DISPLAY D Create Decision Tree classifier with maximum depth = D Train classifier using X_train and y_train CREATE tree visualization using: - trained classifier - training data - target name = "Algorithm" - feature names = [day of week, period of the day, consumption, light intensity, CO2] - class names = [ANN, KNN] - sample X Display tree visualization Print decision tree rules END FOR

The cleaned data and the identified context are sent to a Forecasting agent that performs forecasts either with artificial neural network (ANN) or k-nearest neighbors (KNN). These two algorithms are considered in this paper as previous work conclude the use of these algorithms for training and predictions tasks as the most suitable in the context of this paper [24]. Although this paper addresses. Although previous work provides similar case studies that involve forecasting activities [24], the innovation of this paper is detailed according to the following agents: Context identification agent and Learning agent. A Schedule agent synchronizes the predictions of the consumption of the building to periods to five minutes. Moreover, this schedule runs the predictions while differentiating the different contexts defined previously by the decision tree rules. The forecasting algorithms’ performance is evaluated with the support of an error metrics known as SMAPE as displayed in Eq. (1) which stands for Symmetric Mean Absolute Percentage Error. This metric was used in this paper to compare it with the error calculated in previous publications of the authors of this paper [24]. The ANN model is structured with an input layer containing twelve neurons, followed by two hidden layers containing 32 neurons, and finally an output layer that contains a single neuron.

Ten of the twelve neurons of the input layer represent past consumptions of the building. Two of the twelve neurons of the input layer represent sensor values (CO2 and light intensity) that occur in the period before the output consumption. The only neuron of the output layer represents the consumption following the periods of the consumption variables of the input layer. The training phase is configured with a learning rate assigned to 0.001 leading to very accurate searches that minimize the loss. The number of epochs is assigned to a total of 200 iterations, although an early stopping procedure is added to finalize the training on the condition that within 10 sequential iterations there are no training improvements. The ANN model is supported by the tensorflow library. The KNN model is configured with the support scikit-learn library and five neighbors to find for the most frequent class for the five training observations with most similar features to each test. The Learning agent has an evaluation mechanism to select an algorithm to perform predictions for the respective period of five minutes. This evaluation is performed for all periods of five minutes of a target week that respect each one of the contexts created previously by the Context identification agent. The reinforcement learning methodology of this paper is based on the multi armed bandit algorithm and the upper confidence bound method. Furthermore, the action criteria of the multi-armed bandit algorithm for this paper consists in selecting the algorithm for forecasting activities that is expected to decrease the forecasting error according to SMAPE metrics as displayed in Eq. (1). The multi-armed bandit algorithm selects, for each slice of 5 min, for each context, the used forecasting model (ANN or KNN). There is no delay or switching cost when changing models.

The action criteria depicted in Eq. (2) relies on the importance of each action and the number of times the action was taken. The configuration of the learning rate and the reward of each action influence the calculation of the importance of each action. The reward can either be assigned to 0 or 1. This relies on whether the algorithm that was selected (ANN or KNN) to perform forecasts resulted respectively in a higher or lower forecasting error than the other algorithm. The Pseudocode of reinforcement learning is presented after Eq. (3).

(1) $$SMAPE=100*\;\frac{1}{n}*\sum_{i=1}^{n}\frac{\left|FC\left(t\right)-AC\left(t\right)\right|}{\left(\left|AC\left(t\right)\right|+\left|FC\left(t\right)\right|\right)\;/\;2}$$

• AC – actual consumption
• FC – forecasted consumption
• t – time period
• n – current moment
  (2) $$A_{F}=\text{max}\left(Qt\left(a\right)\;+\;2*\sqrt{\frac{\text{ln}\left(t\right)}{Nt\left(a\right)}}\right)$$
  (3) Qt(a)=Qt(a)+LR*(R−Qt(a))
• $A_{F}$ – action (selected forecasting algorithm)
• Qt(a) – importance of each action
• LR – learning rate
• R - reward
• t – time period
• Nt(a) – number of times the action was taken

**Table:** 

Pseudocode of reinforcement learning
DEFINE CLASS MultiArmedBandit_UCB: INITIALIZE: set number of actions k = 2 set exploration rate (exp_rate) set learning rate (lr) initialize value estimates = 0 for each action initialize counters (total steps, action counts) load dataset (Consumption, KNN forecast, ANN forecast) initialize reward history and action history FUNCTION chooseAction: IF random number < exp_rate THEN choose random action (exploration) ELSE compute confidence bounds: value + c * sqrt(log(total_time) / (action_count + small_value)) choose action with highest bound RETURN action FUNCTION takeAction(action): increase time counters compute error_KNN = |consumption - KNN forecast| compute error_ANN = |consumption - ANN forecast| IF action correctly selects better model THEN reward = 1 ELSE reward = 0 END IF update estimated value using incremental update: value[action] = value[action] + lr * (reward - value[action]) store total reward and average reward history FUNCTION play(n): REPEAT n times: action = chooseAction() takeAction(action) store action in history END REPEAT MAIN PROGRAM: DEFINE exploration rates and learning rates FOR each exploration rate: FOR each learning rate: create MultiArmedBandit agent play(N) store: actions average reward END FOR

### Method validation

In this section, data of a building is studied for patterns of five minutes from 18 to 24 November 2019. Another aspect that motivated the selection of this sample is to compare the results of this paper with previous results [20]. The results here validated are very relevant in the field of energy consumption forecast in buildings, with 5 min time stamp. Moreover, it gains additional value when providing information for decisions on energy management. More accurate forecast means better informed decisions. One example is the decision of the optimal operation of energy storage to cover peak demand in winter season [21], where, knowing in advance the electricity consumption allows better decisions on battery charge and discharge. Another example is the provision of flexibility by load assets, as in [22]. In this case, knowing in advance the load consumption may allow flexible management of such load for the convenience of electricity grid operator in a specific period due to punctual technical issues.

**Fig. 2:**
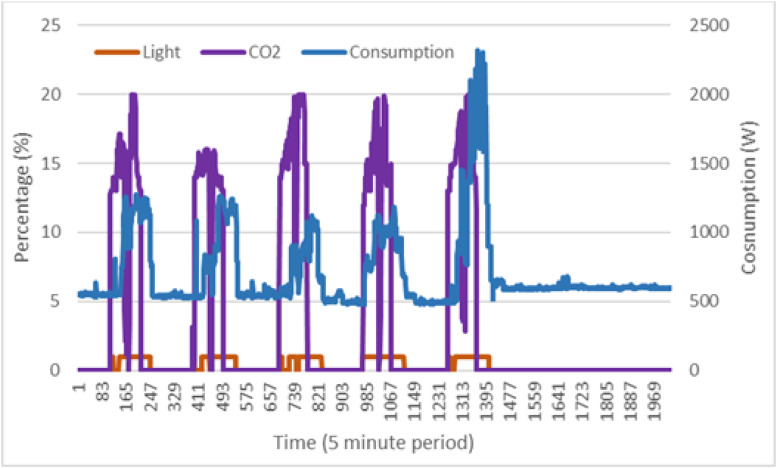
Input parameters of train data (decision tree).

The data sample considered for the decision tree agent studies parameters more useful on the creation of decision rules. Hence, the parameters considered are the following: consumption, CO2, light sensor, allocated period, and the day of the week. The profile of the non-linear features including the consumption, CO2 and a light sensor are analyzed in Fig. 2 for five minutes contexts from 18 to 24 November 2019.

In summary, the data and the related sensors have the following characteristics:

- the dataset duration is from 22nd May 2017 to 17th November 2019 for the training, and from 18th to 24th November 2019 for the test and actual prediction.

- it covers therefore the autumn/winter season in Portugal

- missing data was replaced by average between the closest recent and future values

- the sensors existing in the building and used to provide information to the dataset are: energy consumption (use of previous periods to predict the next one), CO2 levels, and light intensity sensor. In fact, the sensors available include other parameters like temperature, humidity, air conditioning status. However, previous works of the authors proved that the most correlated data for consumption forecast is the one provided by the selected sensors.

The set of patterns is presented for the different days of the week: Monday to Sunday. The first five patterns identify the activity hours on the weekdays. The last two patterns identify the low activity of the weekend. The consumption during activity periods usually has ranges between 700 and 1300 W. However, Friday has more productivity than the other weekdays with a maximum daily consumption higher than 2000 W. The light sensor either shows activity in at least a part of the building (value 1) or no activity at all (value 0).

**Fig. 3:**
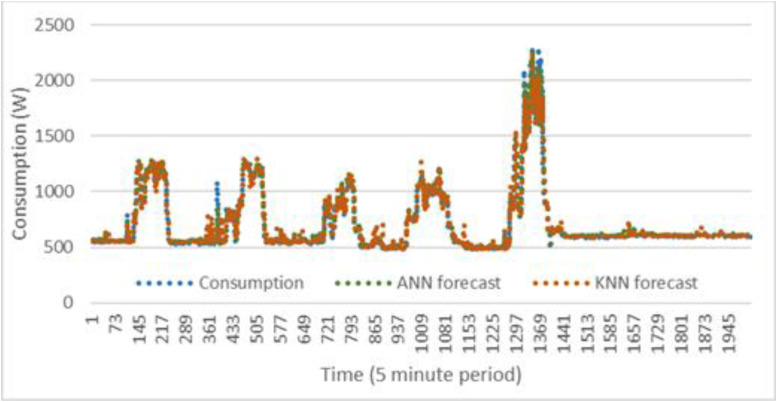
Consumption profile and forecasts from 18 to 24 November 2019.

The learning agents evaluate the algorithm (ANN or KNN) that should perform forecasts for each period of five minutes. Therefore, the real profile consumption and respective forecasts are required to be analyzed for all periods of five minutes. The consumption profile is depicted along with the respective forecasts provided by ANN and KNN algorithms in Fig. 3.

The evaluation of the algorithm (ANN or KNN) to perform forecasts for each period of five minutes uses the multi-armed bandit algorithm. This is proceeded accordingly to the upper confidence method involving the action criteria, the reward calculation and the importance of each action that are acknowledged in the methodology section.

As an example, we show the reinforcement learning evaluation provided by the upper confidence bound learning method according to four contexts (SC1, SC2, SC3, SC4).

Starting with the application of the decision tree, the accuracy and the quantity of contexts of the decision tree are presented in Table 1 for different depth parameterizations.

**Table 1:** Decision tree accuracy and quantity of contexts associated with depth parameterization.

Depth	2	3	4	5	6	7
Accuracy (%)	66.96	66.96	66.96	67.86	71.43	70.54
#Contexts	4	8	15	27	46	75

**Fig. 4:**
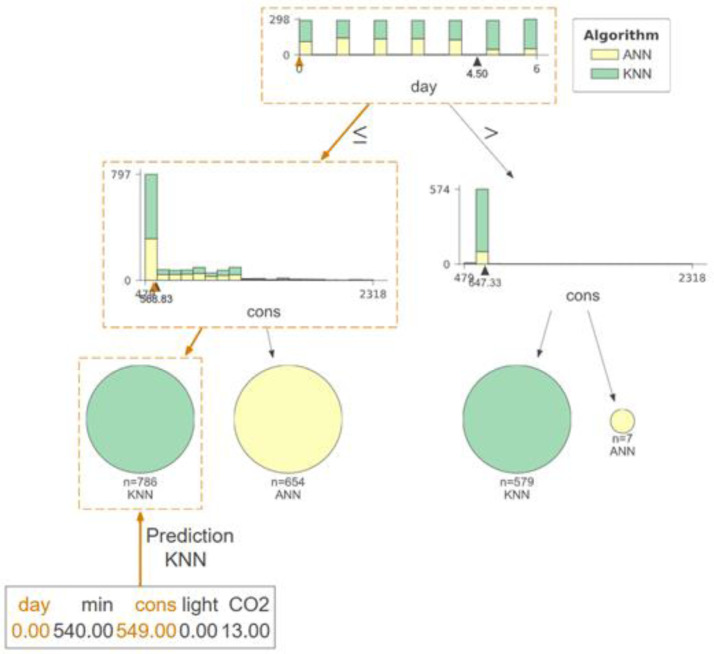
Decision tree for a depth parameterization assigned to 2.

Table 1 shows that alterations in the depth value between 2 and 4 do not change the value of the accuracy assigned to 66.96%. The prediction accuracy is improved changing the depth value from 4 to 5 or from 4 to 6 about 0.90% and 4.47%, respectively. A simple rules elaboration is in Fig. 4 for a depth parameterization assigned to 2. As can be seen in Fig. 4 and in List 1, the labels of data used as input for the decision tree are: day of the week, consumption of the building, minute tag, light intensity, and CO2.

The rules in Fig. 4 present very simple logics involving the day of the week and consumption profile ranges. Two relevant contexts are identified including a) weekday from Monday to Friday and consumption ranges below or equal to 568.833 W or b) weekday from Monday to Friday and consumption ranges larger than 568.833 W. The elaboration of simple rules for a decision tree depth assigned to 2 is represented in List 1.

|— day of the week ≤ 4.50

| |— consumption of the building ≤ 568.83

| |— consumption of the building > 568.83

|— day of the week > 4.50

| |— consumption of the building ≤ 647.33

| |— consumption of the building > 647.33

List 1. Decision rules elaboration for a depth parameterization assigned with the value two

The two restrictions a) and b) are used as basis to identify four contexts (SC1, SC2, SC3, SC4) deduced from more complex rules. Table 2 shows the rules restriction that were considered to create each context.

**Table 2:** Contexts and rules generated from the decision tree training.

Context	Rule
SC1	day of the week ≤ 4.50 AND consumption of the building ≤ 568.83
SC2	day of the week ≤ 4.50 AND consumption of the building > 568.83
SC3	day of the week > 4.50 AND consumption of the building ≤ 568.83
SC4	day of the week > 4.50 AND consumption of the building > 568.83

This complexity considers a depth parameterization assigned to 6 due to this parameterization resulting in more accurate predictions. The rules elaboration with this complexity are represented in List 2 presenting a total of 46 contexts. The factors involved in these rules include the day of the week, consumption profile, CO2 and the allocated period. The selections of contexts SC1, SC2, SC3 and SC4 respect the restrictions presented in a) and b) separating contexts presenting small from large occurrences.

The learning phase analyzes the average of rewards and the history of actions for five minutes contexts for all exploration and exploitation rates from 0.1 to 0.9 considering the upper confidence bound learning method.

|— day of the week ≤ 4.50

| |— consumption of the building ≤ 568.83

| | |— CO2 ≤ 13.35

| | | |— consumption of the building ≤ 540.83

| | | | |— consumption of the building ≤ 485.83

| | | | | |— time period ≤ 417.50

| | | | | |— time period > 417.50

| | | | |— consumption of the building > 485.83

| | | | | |— time period ≤ 1242.50

| | | | | |— time period > 1242.50

| | |…

List 2. Decision rules elaboration for a depth parameterization assigned with value six

**Fig. 5:**
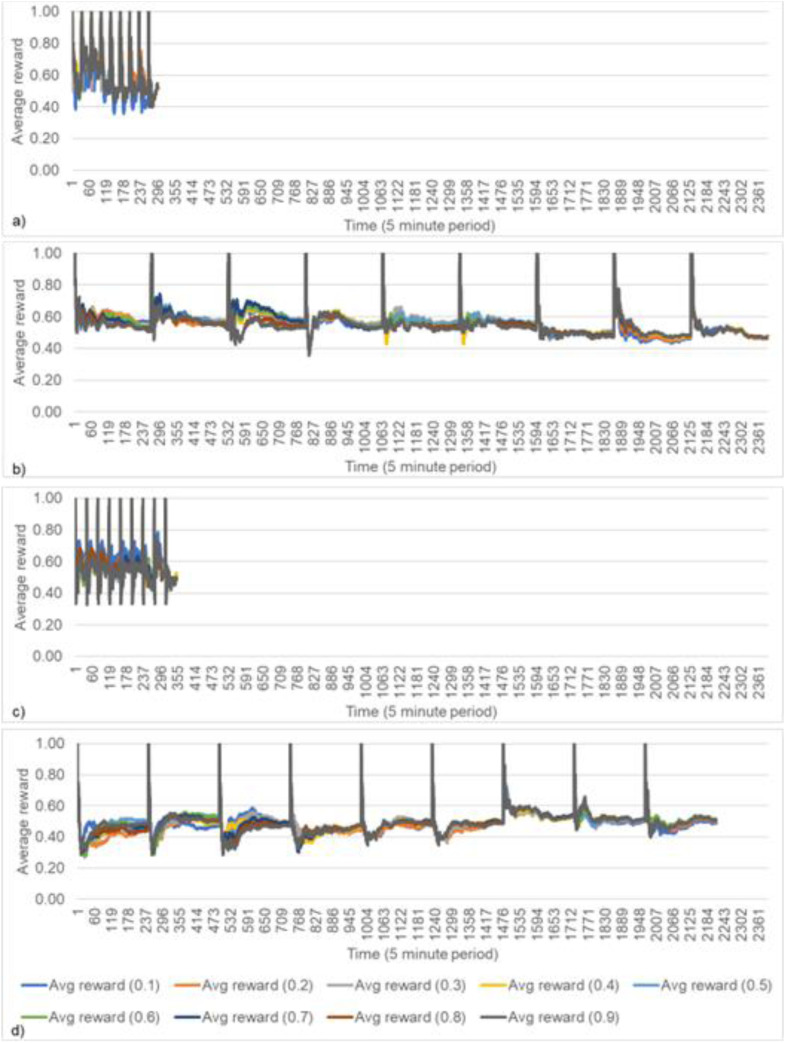
Average of rewards for contexts SC1, SC2, SC3, and SC4 for exploration and exploitation rates from 0.1 to 0.9.

The selection of actions in five minutes context corresponds to the forecasting algorithm selection, either KNN (labeled a 0) or ANN (labeled as 1). Five minutes rewards are assigned to 0 or 1 meaning the selection of a forecasting algorithm resulting respectively in higher or lower forecasting error. The average rewards are presented for all four contexts (SC1, SC2, SC3, SC4) and for all exploration and exploitation rates in Fig. 5. The library used for reinforcement learning is presented in [23].

**Fig. 6:**
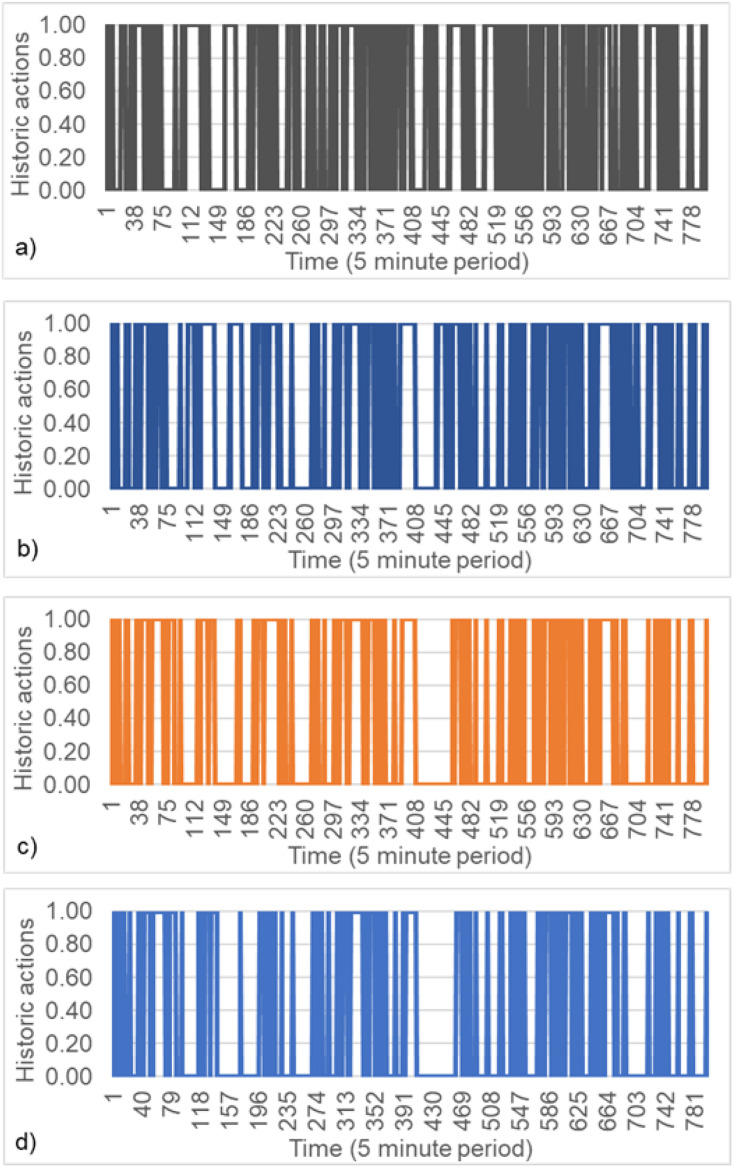
Historic of actions for context SC2 and exploitation rate assigned to 0.9, 0.7, 0.2 and 0.1, respectively in a), b), c) and d) labeling the forecasting decision for five minutes contexts as 0 (KNN) or 1 (ANN).

The historic of actions is illustrated for context SC2 and for four exploitation rates parameterized with values 0.9, 0.7, 0.2 and 0.1 labeled respectively in a), b), c) and d) in Fig. 6. These exploitation rates were the cases identified to result in larger average rewards as identified in Fig. 5.

The historic of actions presented in Fig. 6 shows two possible behaviors: to evaluate repeatedly as KNN or to exchange forecasting algorithm selections between KNN and ANN. Increasing the exploration rate results in a faster acquisition of knowledge of each forecasting algorithm selection. Therefore, the decrease of the exploration rate motivates us to evaluate more five minutes periods as KNN as evidenced between 408 and 445 long sequences of five minutes when comparing scenarios b) with c) in Fig. 6 with exploitation rates of respectively 0.7 and 0.2.

**Fig. 7:**
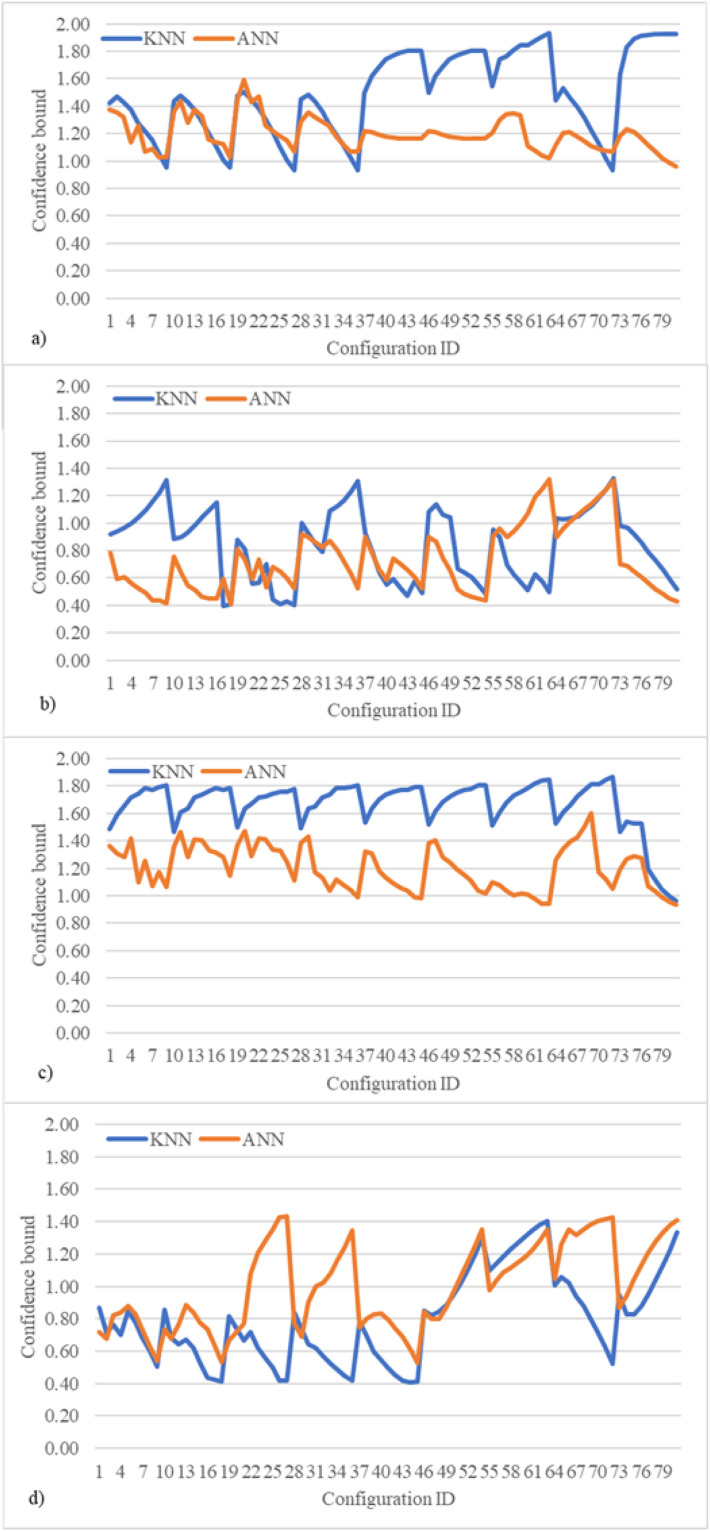
Confidence bound for contexts SC1 (a), SC2 (b), SC3 (c) and SC4 (d) obtained from upper confidence bound learning method labeling KNN and ANN.

The confidence bound obtained from the upper confidence bound learning method is presented for different configurations and for contexts SC1, SC2, SC3 and SC4 in Fig. 7 labeled respectively as a), b), c) and d). The confidence bounds are classified as KNN and ANN representing the trust of each forecasting algorithm. Furthermore, the confidence bound varies in Fig. 7 for different contexts (SC1, SC2, SC3, SC4) for a total of 81 configurations. These configurations represent quantitative changes to the exploration and exploitation rates (from 0.1 to 0.9). The confidence bounds are marked respectively in blue and orange in Fig. 7.

Although on overall all parameterization value combinations benefit more KNN than ANN, context identifications may benefit on some scenarios more ANN than KNN as evidenced in Fig. 7. For example, scenario d) benefits more ANN than KNN for most configurations respecting explorations rates higher than 0.3 or exploration rates equal to 0.3 and exploitation rates equal or higher than 0.3. Using context is advantageous as it reaches confidence bound values higher than one as evidenced in the four scenarios in Fig. 7.

Table 3 present forecasting errors with SMAPE evaluation metrics and reinforcement learning application for all exploration and exploitation rates and for context SC3. The forecasting accuracies of KNN and ANN with no reinforcement learning application is presented as well. The SMAPE metrics with reinforcement learning application shows low forecast errors for context SC3 with ranges between 3.50 and 4.09%. Moreover, reinforcement learning application proves advantageous as demonstrated by lower forecasting SMAPE errors when compared to the use of KNN and ANN algorithm with no reinforcement learning enhancement.

**Table 3:** SMAPE forecast errors in SC3 context (%).

Exp	0.1	0.2	0.3	0.4	0.5	0.6	0.7	0.8	0.9
0.1	3.69	3.75	3.75	3.69	3.83	3.82	3.83	3.78	3.83
0.2	3.65	3.63	3.73	3.68	3.67	3.72	3.63	3.60	3.64
0.3	3.52	3.50	3.60	3.55	3.55	3.59	3.51	3.51	3.55
0.4	3.68	3.71	3.80	3.71	3.76	3.65	3.65	3.65	3.67
0.5	3.63	3.69	3.60	3.60	3.60	3.60	3.60	3.64	3.64
0.6	3.63	3.69	3.60	3.60	3.60	3.60	3.60	3.64	3.64
0.7	3.85	3.90	3.90	3.90	3.88	3.88	3.94	3.98	3.98
0.8	3.98	4.03	4.03	4.07	4.07	4.07	4.07	4.07	4.09
0.9	3.89	3.89	3.89	3.89	3.99	3.99	3.99	3.99	3.99

The SMAPE errors obtained in SC3 context were 3.54% with KNN and 4.79% with ANN. The obtained SMAPE forecasting errors in SC4 context were 4.91% with KNN and 4.54% with ANN. SC3 shows four possible parameterizations with lower forecasting error including the best scenario resulting in 3.50% with exploration rate 0.3 and exploitation rate 0.2. In [20], the authors have reached a method setting resulting in 4.56% of SMAPE. In the present paper, the lower SMAPE is 4.47%.

## Limitations

Although the methodology section of this paper is rather complete, the authors recognize some limitations to be addressed in the future. Firstly, the set of algorithms of the forecasting agent are limited to the Artificial Neural Networks (ANN) and K-Nearest Neighbors (KNN). Hence, the authors intend to reference and test additional forecasting algorithms in future papers including Linear Regression (LR), Support Vector Machine (SVM), Random Forest (RF), and eXtreme Gradient Boosting (XGB). Another limitation in the reinforcement learning area since only the multi-armed bandit algorithm is used to evaluate the most viable forecasting algorithm in different periods of five minutes. Other algorithms of the reinforcement learning area will be considered in future research including q-learning, REINFORCE and Proximal Policy Optimization.

## CRediT author statement

**Daniel Ramos:** Writing- Original draft preparation, Data curation, Investigation, Conceptualization, Methodology, Software. **Pedro Faria:** Conceptualization, Methodology, Validity tests, Data curation, Writing- Original draft preparation, Supervision. **Pedro Campos:** Writing- Reviewing and Editing, Validation, Supervision. **Zita Vale:** Conceptualization, Methodology, Writing- Reviewing and Editing.

## Ethics statements

Not applicable.

## Declaration of competing interest

The authors declare that they have no known competing financial interests or personal relationships that could have appeared to influence the work reported in this paper.

## Data Availability

Data will be made available on request.
